# Comparison of College Students’ Energy Expenditure, Physical Activity, and Enjoyment during Exergaming and Traditional Exercise

**DOI:** 10.3390/jcm7110433

**Published:** 2018-11-10

**Authors:** Daniel J. McDonough, Zachary C. Pope, Nan Zeng, Jung Eun Lee, Zan Gao

**Affiliations:** 1 School of Kinesiology, University of Minnesota-Twin Cities, Minneapolis, MN 55455, USA; mcdo0785@umn.edu; 2 School of Public Health, Division of Epidemiology & Community Health, University of Minnesota-Twin Cities, Minneapolis, MN 55455, USA; popex157@umn.edu; 3 Department of Food Science and Human Nutrition, Colorado State University, Fort Collins, CO 80526, USA; nan.zeng@colostate.edu; 4 College of Education and Human Service Professions, University of Minnesota-Duluth, Duluth, MN 55812, USA; junelee@d.umn.edu

**Keywords:** Active video games, Acute Exercise, Exergaming, Physical activity, Physical fitness

## Abstract

This study evaluated the effects of exergaming on college students’ energy expenditure (EE), moderate-to-vigorous physical activity (MVPA), light physical activity (LPA), rating of perceived exertion (RPE), and enjoyment compared to traditional treadmill exercise, and sex differences. Sixty college students (30 female; $\bar{X}$_age_ = 23.6 ± 4.1 years) completed three 20-min exercise sessions on Xbox 360 Kinect Just Dance (Microsoft, Redmond, WA, USA), Xbox 360 Kinect Reflex Ridge (Microsoft, Redmond, WA, USA), and treadmill walking. Their EE and PA were assessed by ActiGraph accelerometers (ActiGraph Co.; Pensacola, FL, USA); RPE every four min; enjoyment via an established scale. Significant exercise-type by sex interaction effects were observed for RPE (*p* < 0.01): females reported significantly lower RPE during exergaming sessions but significantly higher RPE during treadmill walking. Results revealed significant main effects for all outcomes between exercise sessions (all *p* < 0.01): treadmill walking resulted in significantly higher metabolic equivalents (METs), MVPA, and EE (*p* < 0 .01), yet lower LPA (*p* < 0.01), compared to the two exergaming sessions. Participants’ RPE was significantly higher during treadmill walking than during exergaming sessions, with exergaming eliciting significantly higher enjoyment (all *p* < 0.01). College students find exergaming more enjoyable and report lower RPE compared to traditional treadmill exercise, though not yet matching the moderate physiological intensity level.

## 1. Introduction

Over one-third (approximately 37%) of the USA population is obese (body mass index (BMI) ≥ 30 kg/m^2^)—A condition associated with greater risk of chronic diseases like type 2 diabetes, stroke, and heart disease [1]. Physical inactivity (time awake spent sitting or lying; metabolic equivalents (METs) ≤ 1.5) has been identified as a risk factor, independent of physical activity (PA) [2,3]. Of concern is the fact obesity is affecting younger generations, with 32.3% of young adults in the USA aged 20–39 years classified as obese [4]. In particular, a cohort within this age group demonstrating increased risk for overweight and obesity are college students, as PA and nutritional decisions are often being made autonomously for the first time [5,6]. In addition, time spent studying or in class, the need to work to support the cost of schooling, and the high usage of technology like smartphones (owned by 83% of college-aged adults) induce sedentary behavior and limit time available for regular PA participation [6,7]. As one in three young adults in the USA attends college, college campuses may represent an ideal environment within which to promote PA participation. Given young adults’ interest in technology [8], potential technology-based PA intervention strategies should be investigated for their ability in promoting physiological and psychological health.

Exergaming refers to video games that require bodily movement during gameplay [9]. This PA modality has shown promise for promoting PA in various populations [10,11,12,13]. In the USA, 70% of male and 50% of female young adults between the ages of 18 and 29 report playing video games consistently [14]. Among the most popular gaming devices is Microsoft’s Xbox 360 (Microsoft, Redmond, WA, USA), with 85 million units sold worldwide from 2005 to 2017 [15]. Moreover, the Xbox 360’s Kinect unit—A motion sensing device used to capture bodily movement and promote PA—sold approximately 25 million units in 2013 [16]. Given young adults’ interest in video game play [14], exergaming may be an effective method of promoting PA and improving physiological and psychological health outcomes among college students.

Literature has observed exergaming to elicit light-to-moderate PA intensities—Similar to that of brisk walking—As quantified by PA, energy expenditure (EE), and rating of perceived exertion (RPE) measurements [17,18]. Notably, however, previous literature has also indicated the enjoyable nature of exergaming to lower the RPE one experiences during PA at a moderate-to-vigorous intensity [19,20,21,22,23]. As such, college students may be able to participate at PA intensities necessary to produce health benefits, without perceiving a high level of exertion—Potentially increasing not only the duration of a single PA session and resulting in greater EE, but also potentially improving the likelihood of long-term PA participation. Furthermore, exergaming’s effect on EE is also important to consider and is dependent on the nature of the respective exergames. Indeed, some exergames are designed to be more physically demanding—Particularly those requiring total body movement (e.g., Kinect Just Dance (Microsoft, Redmond, WA, USA)) [21]. For example, a significantly increased EE and heart rate (HR) has been observed in adolescent boys and girls who engaged in exergames requiring whole body movement compared to those only requiring movement of the upper body [21]. Additionally, a recent meta-analysis of 35 studies examining the health-related outcomes of exergame play in children and adolescents indicated that exergame play resulted in increased METs and PA when compared to sedentary video game play [24]—Similar to other reviews on the topic [11,25]. Yet, while exergaming has shown promise for increasing EE and enjoyment, investigations have primarily focused on children and adolescents [23,26], though some have examined young adults [27]. Information on the physiological and psychological effects of exergaming compared to traditional exercise among college-aged individuals is scarce, with no known investigations comparing two different exergames (i.e., dance, adventure) to traditional exercise.

Sex also appears to play a role in PA participation. For example, males have long been observed to be more physically active than females after the age of 10–12 years—A trend also observed in adult men and women [28]. Females often shy away from PA participation as a whole for fear of being perceived as too masculine [29]. Indeed, compared to males, females diet more often and are observed to have less body satisfaction—likely attributed to the “ideal” female body shape created by contemporary societal norms [30]. Additionally, motivation and enjoyment of PA may play a major factor in PA participation—Especially among college-aged females. For example, female college students are motivated to overcome body dissatisfaction and manage their weight through exercise (i.e., extrinsic motivation)—Possibly decreasing their enjoyment for PA and negatively affecting long-term PA adherence [30]. However, given that college-aged females tend to believe that acute, interval-type bouts of exercise (e.g., exergaming) expend more energy than continuous exercise (e.g., treadmill walking) and find this type of exercise more effective for weight loss, they may find exergaming as a more enjoyable PA modality [31]. Thus, it is important to identify PA modalities that not only provide physiological and psychological health benefits, but are enjoyable enough to attenuate the differences between sex for PA. Given these differences, the current study also sought to investigate the effect of sex on the outcomes.

Given the preceding literature review and the limitations of previous studies, this cross-sectional study had two main purposes: (1) examine the effect of two different exergames on EE, PA duration (minutes of light PA (LPA) and moderate-to-vigorous PA (MVPA)), RPE, and enjoyment compared to traditional treadmill walking; and (2) evaluate the effect of sex on these outcomes. The following hypotheses were proposed: (1) it was hypothesized that the two exergaming sessions would elicit similar EE and MVPA duration compared to the treadmill walking session; (2) it was hypothesized that the two exergaming sessions would result in greater enjoyment and lower RPE than the treadmill walking session; and (3) it was hypothesized that females would report greater enjoyment and lower RPE and have greater EE and time in MVPA than males during the exergaming conditions. Identification of the effects of exergaming on college students’ PA and psychosocial outcomes will help establish effective PA intervention strategies for college students and may help combat the increase in obesity and decline in PA participation in this population.

## 2. Materials and Methods

Data were collected in Fall 2015 and Spring 2016 (Table S1). Participants were recruited via flyers posted around the University and word of mouth. The sample consisted of 60 college students (30 female; 39 non-Hispanic white; Mean _age_ = 23.6 ± 4.1 years; Mean _BMI_ = 23.9 ± 4.0) who volunteered to complete three separate 20-min exercise sessions in a randomized fashion during a single laboratory visit: (1) Xbox 360 Kinect Just Dance; (2) Xbox 360 Kinect Reflex Ridge (Microsoft, Redmond, WA, USA); and (3) treadmill walking at 4.0 mph. 100% of those screened completed the study, 52% of which reported having previous experience with exergaming, with an average skill level of “fair.” University of Minnesota Institutional Review Board Approval (1502M62327) and written informed consent were obtained before data collection. Further, all procedures performed with participants were in accordance with the ethical standards of the Institution and/or national research committee and with the Declaration of Helsinki and its later amendments or comparable ethical standards [32]. Participants were compensated with a $20 ClinCard^®^ (Greenphire Inc., Philadelphia, PA, USA) upon successful completion of the study.

Trained research assistants measured height to the nearest half-centimeter using a Seca stadiometer (Seca, Chino, CA, USA) after which weight and body fat percentage was evaluated via bioelectrical impedance using the Tanita BC-558 IRONMAN^®^ Segmental Body Composition Monitor (Tanita, Tokyo, Japan) digital weight scale.

PA was measured using ActiGraph wGT3X+ accelerometers (ActiGraph Co.; Pensacola, FL, USA)—A valid and reliable measure of PA in children and adults [28]. Accelerometers were worn on an elastic strap during the entirety of the session at the right hip, just above the right superior iliac crest. Because the present study examined acute, intermittent exercise bouts (e.g., exergaming), activity counts were set at a 1-second epoch [33], with empirically-based cut-points used to determine PA intensity for adults: (a) 0–2690 counts/minute = LPA; (b) 2691–6166 counts/minute = moderate PA; (c) 6167–9642 counts/minute = vigorous PA; and (d) ≥9643 counts/minute = very vigorous PA [34,35]. Following completion of the study, each participant’s age, height, weight, race/ethnicity, and sex were imported into ActiLife software (Version 6.13, ActiGraph Co.; Pensacola, FL, USA) to allow for the discernment metabolic equivalents (i.e., a measure of exercise intensity, with one MET equal to 3.5 mL/kg/minute of oxygen consumption) and EE in calories.

RPE was used to evaluate participants’ perceived exercise intensity every 4 min during each of the three 20-min exercise sessions using Borg’s Rate of Perceived Exertion Scale [36]. The Borg RPE Scale has 15 numeric categories (ranging from 6 to 20), representing eight levels of perceived exercise intensity: “no exertion at all” (6), “extremely light” (7–8), “very light” (9–10), “light” (11–12), “somewhat hard” (13–14), “hard or Heavy” (15–16), “extremely hard” (19), and “maximal exertion” (20). The RPE scale has been validated against numerous physiological outcomes (e.g., HR and blood muscle lactate concentrations), and has been found to be reliable in most populations (0.8–0.9) [37].

Enjoyment was measured immediately after each of the 20-min exercise sessions using the Perceived Enjoyment Scale [38]. Specifically, a 5-item assessment of participants’ enjoyment of each exercise session was conducted on a 5-point Likert scale, with 1 = “strongly disagree” and 5 = “strongly agree.” Sample questions included: “I have more fun playing exergames/traditional exercise than doing other things”; “Playing exergames/traditional exercise is the thing I like to do best”; and “I usually prefer to watch rather than play.”

Participants first read and signed the informed consent document, after which they completed a demographic information sheet reporting age, sex, and race/ethnicity. Next, height, weight, and body fat percentage measurements were completed, with individuals dressed in lightweight clothing and without socks. Following the assessment of anthropometric variables, ActiGraph wGT3X+ accelerometers were then accurately placed. Participants completed three separate 20-min exercise sessions on the Xbox 360 Kinect Just Dance, Xbox 360 Kinect Reflex Ridge, and treadmill walking at 4.0 mph in a randomly allocated order.

Xbox 360 Kinect Just Dance and Reflex Ridge were played in single-player, “Competition” mode. Given PA-related sex-norms [30,31,39], these two exergames were chosen to observe if males or females gravitated towards a particular type of exergame (e.g., females gravitated towards Just Dance; males gravitated towards the adventure game). The Sole F63 treadmill (Sole Fitness; Taipei, Taiwan) was used during the treadmill walking session. The treadmill speed was set and maintained at 4.0 mph at a zero-degree incline. Participants were instructed to keep their hands by their sides and swing them as they normally would during traditional walking. Notably, to create a similar exercise environment to that observed in real-world conditions, participants were given the option to either listen to music or watch videos using their phones or tablets during the treadmill exercise. Participants completed a battery of psychosocial evaluations after each exercise session (e.g., Enjoyment and Self-Efficacy Surveys), with 10 min provided between sessions to allow the participants’ blood pressure and RPE to return to baseline (Table 1)—Limiting the potential-carryover effect from one session to the next [40].

Statistical analyses were completed on IBM-SPSS 22.0 (IMN Inc.; Armonk, NY, USA). First, descriptive statistics were calculated for each exercise session. Next, a mixed model analysis of covariance (ANCOVA) with repeated measures (within-subjects factor: exercise session (3 levels); between-subjects factor: sex (2 levels)) and body fat % as the covariate evaluated differences between exercise sessions for MVPA/LPA, EE, enjoyment, and RPE (i.e., main effects), as well as the differences between exercise sessions for these outcomes by sex (i.e., interaction effects). Finally, post hoc Bonferroni analyses were performed to further elucidate differences between the three sessions for the preceding outcomes. The significance level was set at 0.05.

## 3. Results

The final sample with full demographic characteristics is noted in Table 2. Table 3 presents the descriptive characteristics by exercise session. Significant exercise session by sex interaction effects were only observed for RPE, F (2, 171) = 3.82, *p* = 0.02, η^2^ = 0.04, whereby females reported significantly lower RPE than males during both exergaming sessions but significantly higher RPE than males during treadmill walking. Further, significant main effects were revealed for all outcomes between the three exercise sessions (all *p* < 0.01). Specifically, Bonferonni analyses revealed that treadmill walking resulted in significantly higher METs, F (2, 173) = 62.60, p < 0.01, η^2^ = 0.42, and MVPA, F (2, 173) = 69.54, *p* < 0.01, η^2^ = 0.45, yet lower LPA, F (2, 173) = 111.53, *p* < 0.01, η^2^ = 0.56, than the two exergaming sessions. Additionally, Reflex Ridge resulted in significantly higher METs but lower MVPA (both *p* < 0.01) compared to Just Dance. Moreover, there were significant differences for EE, F (2, 164) = 22.30, *p* < 0.01, η^2^ = 0.21, with secondary analyses revealing that the treadmill condition resulted in significantly more calories burned compared to both exergaming conditions (*p* < 0.01). Finally, the two exergaming sessions resulted in significantly greater enjoyment than treadmill walking, F (2, 173) = 33.59, *p* < 0.01, η^2^ = 0.28, while participants’ RPE was significantly higher during treadmill walking compared to both exergaming sessions, F (2, 171) = 12.13, *p* < 0.01, η^2^ = 0.12.

## 4. Discussion

The current study compared PA, EE, RPE, and enjoyment outcomes during two separate exergaming sessions and a traditional treadmill session, as well as the effect of sex on these outcomes. Findings suggest both exergaming sessions to elicit significantly greater enjoyment and decreased RPE compared to the treadmill session, whereas the treadmill session elicited significantly greater time in MVPA, METs, and EE. Moreover, females reported significantly higher RPE during treadmill walking but significantly lower RPE during exergaming sessions.

Findings indicated that treadmill walking resulted in greater mean MVPA and EE than both exergaming sessions—Incongruent with our first hypothesis that both exergaming sessions would elicit similar MVPA duration and EE compared to treadmill walking. Previous research comparing PA and EE during exergaming to that of traditional exercise in children produced results that were in line with our original hypothesis. For example, 5-8 min of exergaming has produced EE similar to that of walking, jogging, and skipping, with the majority of exergames eliciting a PA intensity of 3–6 METs (i.e., moderate-intensity PA), with few exergames resulting in PA intensities of 2.2–7.6 METs (i.e., MVPA) [41,42]. Further, other investigations have reported 25–60% greater EE during exergaming when compared to stationary cycling at matched workloads. Notably, however, the EE and PA findings in the current study are congruent with the RPE observed during treadmill walking. Yet, in an investigation by Kraft et al. [43], similar RPEs among college students were observed between exergaming and traditional exercise—Despite the exergaming sessions resulting in greater HR and EE.

The disagreement between the current study’s findings and past literature may be because past literature has implemented traditional PA conditions like stationary cycle ergometry which is not consistently rigorous—Allowing participants to fluctuate exercise intensity as they control the cycling speed. Contrast this methodology with the treadmill walking condition employed in the present study. Indeed, treadmill speed was set at speed of 4.0 mph—Requiring participants to maintain this exact intensity throughout the entirety of the 20-min session no matter how difficult they perceived the treadmill walking to be. Moreover, it is also notable that stationary cycle ergometry relies primarily on the quadriceps muscles whereas the treadmill uses all large muscles of the body. Literature states that the body’s volume of oxygen consumption is approximately 10–15% higher during walking exercise versus biking exercise [44]. Finally, the difference in type of exercise between PA sessions for exergaming and treadmill sessions may have played a role as well. Indeed, the intermittent nature of the exergaming sessions (i.e., brief periods of rest throughout the exercise bout) allowed for periods of partial recovery during the 20-min exercise session as opposed to the continuous nature of the treadmill session which required constant exertion throughout the entirety of the session—Thereby increasing PA, EE, and RPE [45]. Overall, the preceding factors likely explain the significantly higher MVPA, EE, and RPE observed during the treadmill walking condition versus the two exergaming conditions in the present study and why these findings differ from previous research.

Our second hypothesis was that the exergaming conditions would result in significantly greater enjoyment and lower RPE compared to traditional exercise. Findings were congruent with this hypothesis as both exergaming sessions demonstrated significantly greater enjoyment and significantly lower RPE when compared to treadmill walking. Empirical data in previous literature examining exergaming-related enjoyment among college students is sparse and the available data are mixed. A study by Garn et al. examined college students’ enjoyment of five different Wii Fit games, observing that only the obese participants enjoyed exergaming as compared to traditional treadmill walking [27], whereas Barkley et al. found no BMI difference in enjoyment when comparing exergaming to treadmill walking [7]. Indeed, the type of exercise being performed may play a significant role in perceived enjoyment of exercise. For instance, Bartlett and colleagues found that acute bouts of interval-type, aerobic exercise (e.g., exergaming) produced significantly greater perceived enjoyment than continuous, steady-state, aerobic exercise (e.g., treadmill walking)—Likely due to the varied nature of the interval exercise as opposed to the “boring,” monotonous nature of the continuous exercise; thereby increasing exercise reinforcement and enjoyment [45]. This likely explains the lower RPE reported in the present study for both exergaming conditions compared to the treadmill condition, given that enjoyment and RPE are negatively related to one another (i.e., higher enjoyment, lower RPE) [46]. However, intensity differences between exercise sessions (i.e., exergaming vs treadmill exercise may have also affected RPE, as previous literature has demonstrated higher intensity PA to elicit significantly higher RPE [45]. More practically, females in this study were, on average, shorter than males. Thus, treadmill walking at 4.0 mph for females in this study may have required a brisker walk given our female participants’ shorter stride length vs. the taller male participants—Increasing both the real and perceived exertion of female participants.

Our third hypothesis was supported in that females were found to enjoy exergaming more than males. This finding supports numerous PA literature indicating sex plays a significant role in the PA enjoyment [47,48]. For example, an investigation by Gao et al. indicated that although elementary students enjoyed dance-based exergaming significantly more than traditional tag games, girls demonstrated significantly greater enjoyment than boys during dance-based exergames [39]. The researchers attributed this finding to the girls interpreting and accepting the dance-based exergaming (DDR) as a traditionally feminine activity—Congruent with sex norms for PA [48]. This may explain the lower RPE reported by females during the exergame conditions and the higher RPE during the treadmill condition, as enjoyment and RPE are negatively related to one another [46]. Contrary to these findings, previous literature has reported no sex differences in enjoyment between different exergaming and traditional PA conditions. Indeed, mixed results in the literature examining exergaming-related enjoyment by sex may be influenced by the type of exergames implemented as, for example, males may gravitate towards exercises that are aggressive and masculine in nature [39], resulting in lower enjoyment for other exergames perceived as more feminine, such as DDR or Kinect Just Dance. This may have been the case in the current study as females had a particular affinity for Xbox 360 Just Dance. However, further research is needed to assess college students’ exergaming-related enjoyment, especially between sexes.

Although the current study’s strengths lie in its sample of college students—A population sparse in exergaming research—And assessing both physiological and psychological outcomes of two different exergames compared to traditional PA with random order of the three sessions, this study is not without limitations. For instance, the cross-sectional study design called for each participant to undergo each PA session for only a single, 20-min trial. Thus, it cannot be concluded that the enjoyment, and therefore long-term enjoyment and adherence, to such PA would last over time. Therefore, future studies might implement an exergaming intervention and assess enjoyment, health outcomes, and intervention adherence over longer courses of time. Moreover, participants’ previous PA levels were not accounted for, which may have had some influence on the assessed outcomes and future studies may therefore include an assessment of habitual PA in addition to employing this study’s methodology. Indeed, the sample in the present study included only healthy, normal weight participants (i.e, BMI 18.5–24.9 kg/m^2^) and therefore, results may not be generalized to obese individuals. Additionally, though statistical significance was reached for RPE in the present study, mean score differences (i.e., “very light” (9–10); “light” (11–12)) may not be practically significant for long-term PA behavior change. Thus, results for RPE should be interpreted with caution. Lastly, there is the possibility of a cohort effect due to the cross-sectional nature of this study. Specifically, the cohort of college students used in the present study (i.e., young adults who frequently engage in video game play) likely grew up playing video games [14]—Possibly influencing their enjoyment and therefore, RPE, associated with the exergaming conditions. Longitudinal research designs may be used in future research to overcome such effects.

## 5. Conclusions

Findings suggest that playing Kinect-based exergaming has not yet reached the moderate intensity level of fast treadmill walking. Nonetheless, exergaming may increase perceived enjoyment and decrease RPE among college students, especially in females, compared to treadmill exercise which may improve exercise adherence. More study on eliciting greater physiological stimulation during exergaming among college students is needed. With this information, college campuses may consider implementing exergaming stations in their Student Recreation Centers as an attractive and effective option for students to engage in PA. Additionally, based on findings from the current study, students may choose to purchase exergaming devices as an alternative to sedentary-based video games, thereby reducing physical inactivity and promoting enjoyable PA.

## Figures and Tables

**Table 1:** Pre-/post- RPE and blood pressure (BP) scores in a counterbalanced study design.

	RPE		Diastolic BP		Systolic BP	
	Pre	Post	Pre	Post	Pre	Post
Treadmill walking	6.33	13.12	72.14	78.93	117.83	137.42
Kinect Reflex Ridge	6.19	12.54	72.97	76.16	120.27	132.38
Kinect Just Dance	6.49	10.78	73.64	74.98	118.27	128.41

Note: All scores reported as mean values. Participants were randomized into one of three exercise sessions and underwent all three sessions in a counterbalanced study design. 10 min of complete rest was given to participants between each session to allow RPE and BP to return to approximately baseline levels. Abbreviations: RPE, rating of perceived exertion; BP, blood pressure.

**Table 2:** Demographic characteristics of participants.

Characteristic	Mean	SD
Age (years)	23.6	4.1
Height (cm)	171.8	8.2
Weight (kg)	70.7	15.2
^1^ Body fat %	21.3	8.2
Waist Circumference (cm)	80.5	10.2
BMI (kg/m^2^)	23.9	4.0

^1^ Covariate for statistical analyses. Abbreviations: BMI, body mass index; SD, standard deviation.

**Table 3:** Descriptive statistics for outcome variables by sex.

	Sex	MVPA (Time)		LPA (Time)		METs		EE (Cals)		Enjoyment		RPE	
		Mean	SD	Mean	SD	Mean	SD	Mean	SD	Mean	SD	Mean	SD
Treadmill walking	Male	18.31 ^1^	5.00	1.69	0.49	6.35 ^1^	2.24	49.15 ^1^	22.64	2.08	0.61	11.33 ^1^	2.22
	Female ^4^	18.79 ^1^	3.83	1.21	1.74	6.30 ^1^	2.00	44.70 ^1^	15.59	2.45	0.69	12.52 ^1^	2.00
Kinect Reflex Ridge	Male	10.25	3.57	9.75 ^3^	3.19	3.78 ^2^	1.49	32.16	16.00	2.76 ^3^	0.61	11.37	2.22
	Female ^4^	9.56	3.12	10.44 ^3^	3.15	3.53 ^2^	1.33	26.20	14.77	2.97 ^3^	0.58	10.41	2.31
Kinect Just Dance	Male	11.96	4.96	8.04 ^3^	4.75	3.29	1.51	29.96	16.00	3.07 ^3^	0.72	10.30	2.34
	Female ^4^	11.04	4.79	8.96 ^3^	4.79	2.75	1.50	24.63	18.01	3.35 ^3^	0.61	9.61	2.28

^1^ Significantly greater than exergaming (*p* < 0.01). ^2^ Significantly greater than the other exergame condition (*p* < 0.01). ^3^ Significantly greater than traditional treadmill walking (*p* < 0.01). ^4^ Females had significantly greater enjoyment and LPA than males. Abbreviations: SD, standard deviation; MVPA, moderate-to-vigorous physical activity; LPA, light physical activity; METs, metabolic equivalents; EE, energy expenditure; Cals, calories; RPE, rate of perceived exertion.

## Supplementary Materials

The following are available online at http://www.mdpi.com/2077-0383/7/11/433/s1, Table S1: Raw_Dataset.
